# Mental illness as a poor prognosis factor in cancer treatment: a review of the difficulties in diagnosing and treating cancer in patients with schizophrenia based on a clinical case

**DOI:** 10.1192/j.eurpsy.2024.1518

**Published:** 2024-08-27

**Authors:** F. Santos Martins, R. Malta

**Affiliations:** ^1^Department of Psychiatry, Centro Hospitalar Universitário S. João (CHUSJ); ^2^ CINTESIS; ^3^Neurosciences and Mental Health Department, Faculdade de Medicina da Universidade do Porto (FMUP), Porto, Portugal

## Abstract

**Introduction:**

Psychiatric patients, and schizophrenia patients in particular, have a lower average life expectancy than the general population, and the high prevalence of physical illnesses contributes to this. In the case of cancer, the incidence seems to be the same or lower compared to the general population, but on the other, the prognosis is frankly worse.

**Objectives:**

We aim to collect evidence about the relationship between cancer and schizophrenia.

**Methods:**

Based on a clinical case of a patient diagnosed with schizophrenia who died of an occult neoplasm, we conducted a narrative review of the literature concerning cancer screening, incidence, mortality and prognosis in patients with schizophrenia.

**Results:**

A 39-year-old male patient was diagnosed with schizophrenia when he was 26 years older. The patient was single, had no children, lived alone and was retired due to his psychiatric condition.

He was admitted to the inpatient ward in January 2023 due to a psychotic relapse after abandoning the prescribed treatment. He remained hospitalised for 14 days, and oral and injectable antipsychotic therapy was reinstated. He was discharged to the psychiatric day hospital unit to promote psychosocial rehabilitation. During this period, he complained about unspecified back pain but did not present any other physical symptoms.

Two months later, he was evaluated by his psychiatrist as an outpatient, and his general condition had become significantly poorer. He had lost over 20 kilograms, his skin was pale, and he complained of back pain. He was referred to an internal medicine consultation. Still, before it was scheduled, he came to the emergency department and was admitted due to digestive bleeding, asthenia and low back pain, with a weight loss of around 25 kilograms.

An abdominal mass was palpated on physical examination, and the chest x-ray showed a “balloon drop” pattern, indicating pulmonary metastases. Two days after being admitted to the internal medicine ward, he died of cardiac arrest.

It is known that the stigma that mentally ill patients suffer often contributes to a delay in diagnosing medical illnesses. In addition, frequent social isolation and poor social family support do not help these patients seek medical care when their physical condition deteriorates. Low adherence to cancer screening and avoidance of routine health care often add to this delay.

**Conclusions:**

As physicians who often deal with individuals with severe mental illnesses, psychiatrists should be extra aware of risk factors and keep a heightened suspicion of medical conditions. They should also promote the adoption of beneficial health behaviours and encourage participation in cancer screening and other relevant health programs.

**Disclosure of Interest:**

None Declared

